# Annotations

**Published:** 1886-10-09

**Authors:** 


### Annotations.

**Low Tenders.** Our attention has been called more than once lately to the considerable fall in the price at which milk tenders are submitted for acceptance by hospital committees. Formerly one shilling per gallon was considered the minimum price which it was safe to pay, and the best-managed hospitals, acting on their experience, refused to accept tenders at this rate. During the last two years the tender-price, in the majority of cases, has been less than one shilling per gallon—a temptation which has been too strong for Poor-Law authorities, but which the best-managed hospitals have steadily declined to believe in. Many institutions have received tenders for various articles during the last month, or will be receiving them during October. We should be glad to receive from the secretaries a comparative statement of the prices at which the various articles were tendered for now and formerly.

**Cheap Milk.** The reduction in the price of milk supplied to hospitals is due, no doubt, to various causes. First of all, experience proves that the quality of the milk is necessarily inferior to the irreducible minimum below which no hospital steward should accept milk for hospital patients. A correspondent of the *Daily Telegraph* points out that this question affects not only the general public and institutions, but more especially dairyfarmers and honest milk vendors in our large towns. Really pure English milk with all its cream cannot profitably be delivered retail from house to house under 5*d.* per quart, yet certain retail vendors offer guaranteed pure milk at 3*d.* per quart, leaving them a good profit. How is this done? Large quantities of unsweetened condensed milk are being imported into this country from abroad, to which water is here added up to the proportion which milk is known to contain in its natural state. This Anglo-foreign mixture offers more than one danger to the consumer. There is no control over the purity of the water added, and no information as to the sanitary condition of the foreign farms whence the milk is supplied. It should be made illegal to add water to condensed milk for purposes of sale, and meanwhile householders and institutions will do well to beware of cheap milk and cheap milk tenders.

**The Dead Hand.** There can be no doubt that the amount of money bequeathed of late years for charitable purposes has steadily declined. We believe inquiry would show that without exception the legacies left to the London hospitals of late years represent a falling off of something like 100 per cent., when compared with the sums they received from this source ten years ago. It would be interesting to inquire why this change has taken place, especially as it is calculated to render it well-nigh impossible to maintain the hospitals in efficiency in the years to come. Inquiry convinces us that in Scotland the reverse is the case, and that more legacies have been left there in recent years than formerly. The munificent bequests to the charitable societies and institutions of Glasgow by the late Mr. Andrew Cunninghame, announced this week, confirm this view. It is stated, for instance, that the Glasgow Royal Infirmary will receive £10,000, the Western Infirmary, £5,000, whilst the Lying-In Hospital, Blind Asylum, Deaf and Dumb Asylum, Lock Hospital, and Eye Infirmary, will each receive £250. May Mr. Cunninghame's example stimulate others to revert to the system of bequests, for it is surely better to give largely in this way to charities than to leave one's money, say, to the strange lawyer called in at the last moment to prepare the will, as a certain lady, the last of her race, recently did.

**Fever Hospitals in Towns.** The Sussex County Hospital at Brighton is ambitious to follow the example of St. Bartholomew's Hospital by building a sanatorium in connection with its management. It appears that there are fever wards in connection with the Sussex County Hospital, and that a sanatorium was to be built for the reception of the infectious convalescents. The Sussex County Hospital is situated within the borough boundaries, and the Town Council, as the Sanitary Authority, has not unnaturally urged the Hospital Committee to give up taking fever patients and the idea of a sanatorium. There is stated to be ample and sufficient hospital accommodation for the reception of the fever patients in the institution under the control of the Local Sanitary Authority. These buildings are situated outside Brighton, and are properly isolated, so that the risk to the inhabitants from an outbreak of fever should not be great, if the entire care of such cases be given over to the Town Council as the Sanitary Authority. It appears that the statutes of the Sussex County Hospital bind the Committee to admit fever cases, but we are glad to know that the Committee have determined to take steps to alter these statutes, with a view to leaving the responsibility of providing accommodation for infectious cases entirely in the hands of the Town Council.

---

A Difference.—The difference between a hill and a pill is that one is hard to get up and the other hard to get down.

